# Primary Intimal (Spindle Cell) Sarcoma of the Heart: A Case Report and Review of the Literature

**DOI:** 10.1155/2013/461815

**Published:** 2013-01-28

**Authors:** A. Ibrahim, A. Luk, P. Singhal, Bo Wan, A. Zavodni, R. J. Cusimano, J. Butany

**Affiliations:** ^1^Department of Pathology, Toronto General Hospital, University Health Network, Toronto, ON, Canada M5G 2C4; ^2^Division of Cardiology, University of Toronto, Toronto, ON, Canada M5T 2S8; ^3^Department of Radiology, Sunnybrook Hospital and University Health Network, North York, ON, Canada M4N 3M5; ^4^Division of Cardiac Surgery, Peter Munk Cardiac Centre, Toronto General Hospital, Toronto, ON, Canada M5G 2C4; ^5^Faculty of Medicine, University of Toronto, Toronto, ON, Canada M5S 1A8

## Abstract

Intimal (spindle cell) sarcomas of the left atrium are extremely rare primary cardiac tumours with three cases reported (Li et al. (2013), Cho et al. (2006), and Modi et al. (2009)). We present a 69-year-old man who first came to medical attention after experiencing abdominal discomfort. He had a 30 lb weight loss apparently due to dieting. He denied any other constitutional symptoms. His symptoms persisted despite a course of antibiotics for presumed diverticulitis. Laboratory values were within normal limits, though the haemoglobin was 131 g/L (normal: 140–180). Subsequent abdominal computed tomography (CT) scan revealed an abdominal wall mass and intracardiac lesion; the cardiac mass was further characterized by transesophageal echo (TEE), magnetic resonance imaging (MRI), and dedicated cardiac CT. TEE revealed a mass attached to the posterolateral wall of the left atrium above the mitral annulus, and the cardiac CT and MRI confirmed the TEE findings. The patient underwent extensive surgical resection and repair of the left side of the heart. Postoperatively, he developed acute renal failure requiring dialysis and reintubation for volume overload. He became acutely hypotensive, developed multiorgan failure, and succumbed to his illness. Histopathologic examination of the left atrial mass showed an intimal sarcoma.

## 1. Introduction

Cardiac tumours may be primary or secondary, with secondary tumours being far more common (100 to 1000 times) [1]. The most common primary site leading to metastatic cardiac involvement is from a pulmonary source [2]. Primary cardiac tumours are rare with an autopsy frequency of 0.001% to 0.03% [1]. Seventy-five percent (75%) of primary cardiac tumours are benign, and nearly half of these are myxomas, with around 10% each of lipoma, papillary fibroelastoma, and rhabdomyoma [1, 3]. About 25% of primary cardiac tumours are malignant [1], and 95% of these are sarcomas; the remaining 5% are lymphomas [1]. The most common cardiac sarcoma is the angiosarcoma (about 37%) [1] while others include undifferentiated sarcoma (24%), malignant fibrous histiocytoma (MFH) (11%–24%), leiomyosarcoma (8%-9%), and osteosarcoma (3%–9%) [1]. Less encountered primary tumours of the heart include rhabdomyosarcoma, liposarcoma, fibrosarcoma, synovial sarcoma, and hemangiopericytoma, with the least reported cardiac tumours being the intimal (spindle cell) sarcomas [1, 4–6].

## 2. Case Presentation

A 69-year-old man was referred for surgical assessment after the findings of an abdominal wall and left atrial (LA) mass. He had mild obstructive lung disease and a 30-pack-year smoking history. He sought medical attention after experiencing abdominal discomfort and had noted a 30 lb weight loss over the last few months, apparently related to dieting. There were no other constitutional symptoms. His symptoms persisted despite a course of antibiotics, for presumed diverticulitis. Physical examination was unremarkable. Laboratory investigations revealed a hemoglobin of 131 g/L (normal: 140–180), with all other values within normal limits. Emergency computed tomographic (CT) scan revealed a 5 × 4 cm heterogenous mass in the left anterior abdominal wall and an intracardiac mass. CT head, chest, and abdomen did not show any other masses.

Two biopsies of the abdominal wall mass were examined, and most of the tissue was necrotic. The conclusion was that a definitive diagnosis was not possible (in spite of the immunohistochemistry) and that the definitive diagnosis was best accomplished following cardiac and abdominal mass resection.

**Figure 1:**
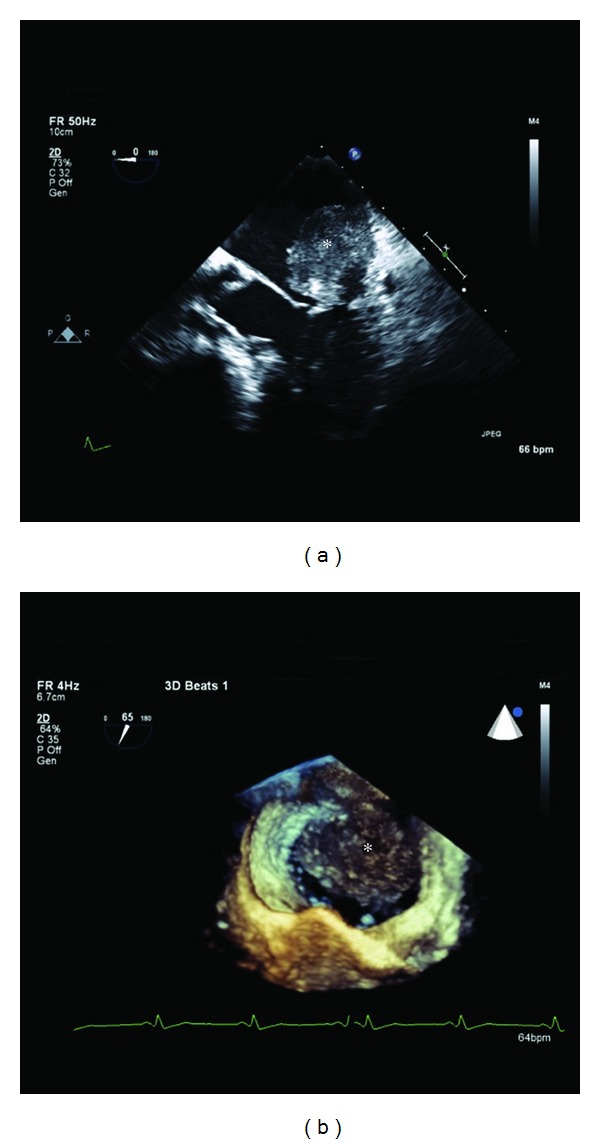
Two-dimensional (a) and magnified three-dimensional (b) transesophageal echocardiogram (TEE) of the left atrium (LA), mitral valve, and left ventricle. A 4.4 × 3.5 cm mass (asterisk) is seen attached to the posterolateral aspect of the LA.

**Figure 2:**
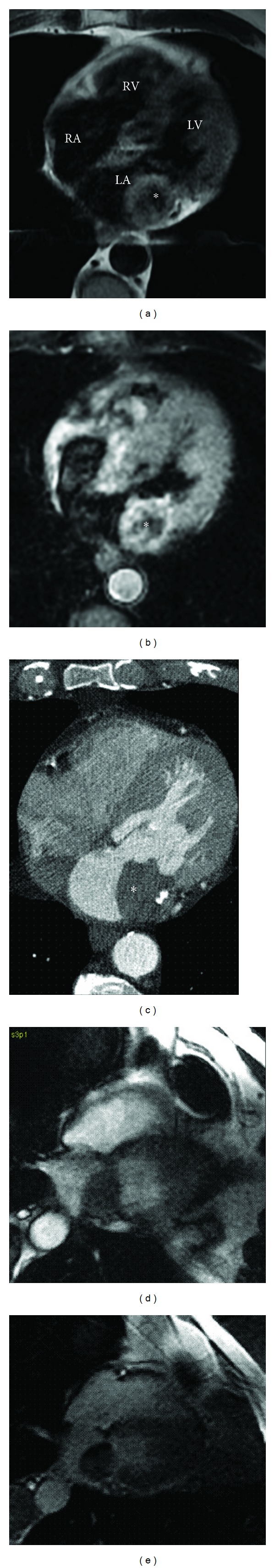
Axial (a) MRI T1- and (b) T2-weighted and (c) CT contrast enhanced imaging and 4-chamber MRI (d) first pass perfusion and (e) late gadolinium enhancement (TI = 350 ms) imaging. MRI T1- and T2-weighted black blood images demonstrate a lobulated mass (asterisk) with central signal drop-out suggestive of punctuate calcification confirmed by CT. CT imaging also suggests that this mass is contiguous with the posterior leaflet of the mitral valve. Dynamic contrast-enhanced MRI demonstrates very minimal first pass perfusion and heterogenous late enhancement consistent with surface thrombus overlying a tumour. (RA) right atrium, (LA) left atrium, (RV) right ventricle, and (LV) left ventricle.

Transesophageal echocardiogram (TEE) revealed a 4.4 × 3.5 cm mass attached to the posterolateral wall of the left atrium, above the mitral annulus. Mean and peak gradients across the mitral inflow were 4 and 12 mmHg, respectively (Figure 1). Cardiac CT and MRI corroborated the TEE findings (Figure 2).

Coronary angiography showed a 90% right coronary artery (RCA) lesion. The patient underwent extensive resection of the LA mass and repair of the left atrium, left ventricle, left ventricular outflow tract, as well as mitral valve replacement and saphenous vein graft to RCA. The postoperative course was complicated by acute renal failure requiring dialysis and reintubation for volume overload. Ten days postoperatively, he became acutely hypotensive, developed multiorgan failure, and succumbed to his illness.

**Figure 3:**
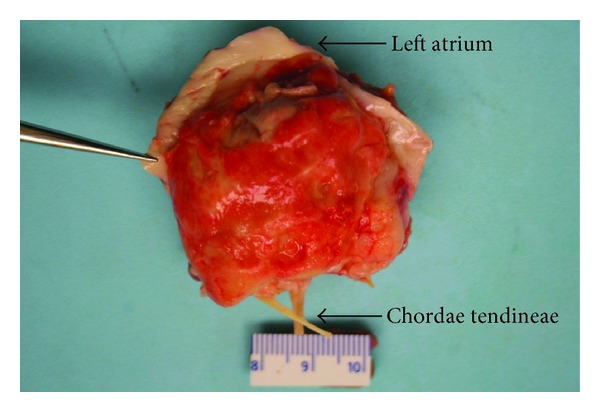
Macroscopic image shows the left atrial mass attached to the posterior wall of the left atrium and the entire posterior mitral leaflet, except for its free margin.

**Figure 4:**
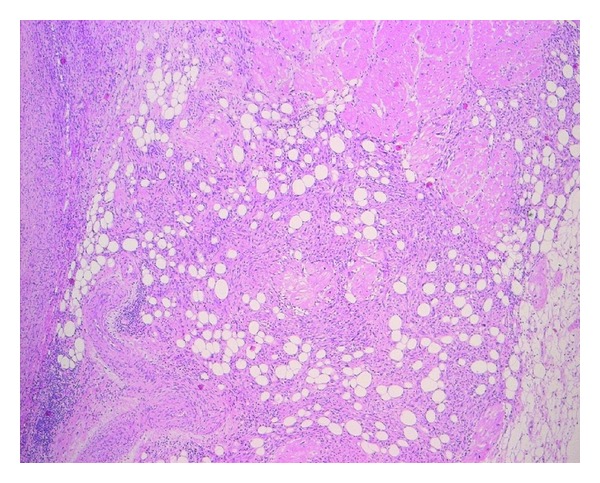
Section shows sheets of densely packed spindle cells infiltrating the myocardium, as well as the overlying epicardial adipose tissue (Stain: H&E, ×5).

**Figure 5:**
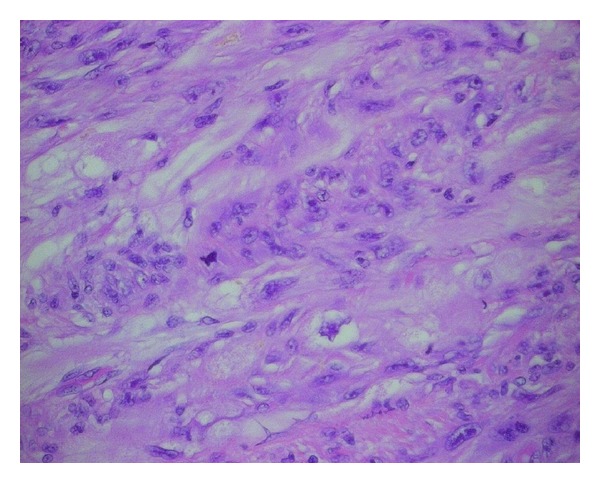
The tumour cells show large, pleomorphic nuclei with prominent nucleoli. An abnormal mitotic figure is identified (Stain: H&E, ×40).

**Figure 6:**
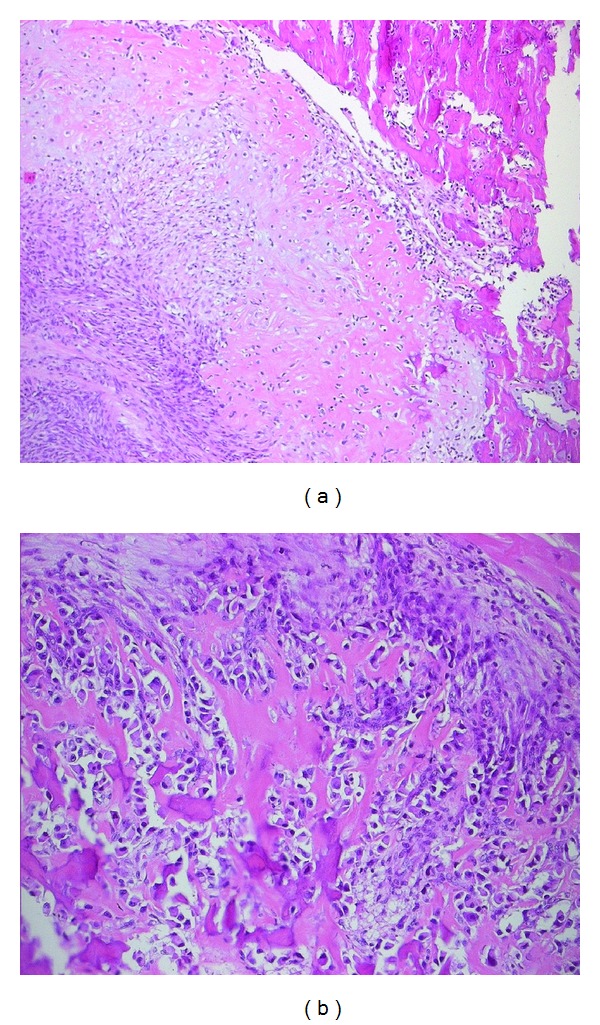
Focal areas of the tumour showed malignant cartilaginous change (a), osteoid and bone formation ((a) Stain: H&E, ×10, (b) Stain: H&E, ×20).

**Figure 7:**
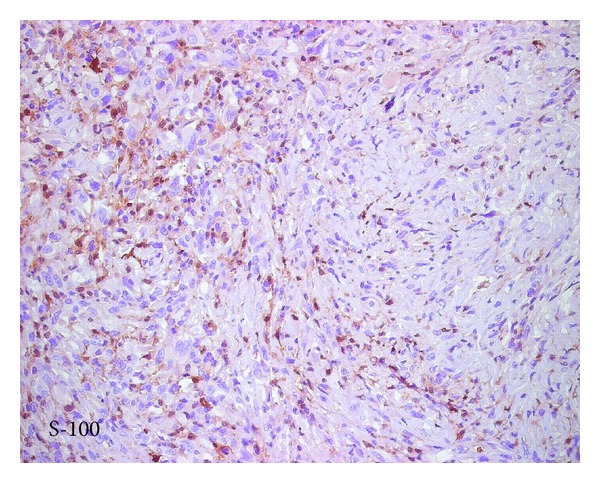
Immunohistochemistry profile shows mild focal positivity for S100.

Macroscopic examination of the cardiac specimen revealed an atrial mass weighing 30.4 grams with a side to side dimension of 4.3 cm, length of up to 4.5 cm, and a thickness of up to 2.9 cm from the atrial endocardium. The mass involved the atrial wall posteriorly and the entire posterior mitral leaflet, except for its free margin (Figure 3). The most protuberant part of the mass was considerably softer in consistency and deeper yellow in color with small darker areas. The cut surface of the mass had a soft to firm grey white appearance. Histology showed sheets of densely packed spindle cells replacing and infiltrating the atrial wall and the mitral leaflet, as well as the epicardial adipose tissue. The tumour cells had large, pleomorphic nuclei with prominent nucleoli (Figure 4). Frequent abnormal mitotic figures were identified (Figure 5). In some areas, the tumour showed malignant cartilaginous and osteoid differentiation (Figure 6). Extensive tumour necrosis was seen, and the free surface had a fairly thick rim of thrombus. Some areas showed tumour extension to the epicardial fat and to the resection margin. The tumour involved the mitral leaflet tissue. Immunohistochemistry had positive staining for vimentin, mild focal positivity to S100 (Figure 7), and negative staining for smooth muscle actin, CD117, CD34, and O13 (CD99). Staining with MDM2 and platelet-derived growth factor (PDGF) was not performed. The left sided cardiac mass was malignant. It showed no evidence of metastasis to the liver, right side of the heart or lungs, and the heart showed no evidence of patent foramen ovale. The morphology of the cardiac tumour compared to the abdominal, and the immunostaining profile, all supported the diagnosis of primary cardiac intimal spindle cell sarcoma.

## 3. Discussion

Columbus, in 1562, first described a tumour mass in the heart [7]. In 1835, Albas, described a cardiac fibroma found at postmortem examination [7–9]. Barnes in 1936 was the first to diagnose a primary tumour of the heart in a living patient using an electrocardiogram and the information of a biopsy taken from a peripheral metastatic lesion. 

Intimal (spindle cell) sarcomas are mesenchymal tumours, more commonly encountered in large arterial blood vessels and extremely rare in the heart, with only 3 cases reported [4–6]. Intimal sarcomas affect the pulmonary artery more than the aorta (165 versus 100 reported patients) [10–12]. They are commonly located in the pulmonary trunk (80%), right or left main pulmonary arteries (50%–70%), or both (40%) [13, 14]. Sometimes, they affect the pulmonary valve or extend into the right ventricular outflow tract. Lung metastases occur in 40% of cases while extrathoracic metastasis occurs in about 20% of cases, involving the kidneys, lymph nodes, brain, and skin [14]. When affecting the aorta, intimal sarcomas are usually located in the abdominal aorta between the celiac trunk and the iliac bifurcation. Less commonly they are located in the descending thoracic aorta. Tumour emboli are common, usually causing distant metastases involving bone, peritoneum, liver, and mesenteric lymph nodes [15]. Macroscopically, intimal (spindle cell) sarcomas form polypoid masses usually attached to the inner surface of the vessel similar to thrombi and sometimes extend distally along the branches of the affected vessel. Osteosarcomatous differentiation can occasionally occur in pulmonary intimal (spindle cell) sarcomas where the tumour may have hard, bony areas. 

Histologically, intimal sarcomas are usually poorly differentiated mesenchymal malignant tumours of fibroblastic or myofibroblastic differentiation, consisting of atypical spindle cells with variable degrees of atypia, mitotic activity, necrosis, and nuclear polymorphism. The tumour may exhibit large myxoid areas and or epithelioid appearance of tumour cells [16, 17]. Tumour cells may resemble leiomyosarcoma and rarely exhibit areas of rhabdomyomatous, angiosarcomatous, or osteosarcomatous differentiation [13, 14, 16, 17]. When an intimal sarcoma arises in the heart, the differential diagnosis includes angiosarcoma or synovial sarcoma. Intimal (spindle cell) sarcomas usually show positive immunoreactivity for vimentin, osteopontin, and MDM2 [10, 18]. Variable positivity was observed for alpha smooth muscle actin, CD117, CD68, p53, and bcl2. Occasionally, the tumour has some positive staining with antibodies against desmin. CD31, CD34, and Factor VIII are typically negative, but may be positive in areas with angiosarcomatous differentiation, indicating the undifferentiated mesenchymal origin of the tumour, with occasional tendency towards differentiation to a specific type of connective tissue.

The prognosis of cardiac primary intimal (spindle cell) sarcomas is generally poor. These tumours are highly aggressive with the mean survival being 3 months to 1 year, although survival of up to 11 years has been reported [6]. Due to the critical nature of the structures involved by the tumour, cardiac tumours can cause significant morbidity and mortality. The effects of a cardiac tumour depend on its anatomical location in the heart, size, invasiveness, friability, and the rate of growth. The most important factor affecting the prognosis of these tumours is the anatomical location in the heart (intracavitary versus intra/extramyocardial growth).

In a recent study using comparative genomic hybridization (CGH), amplifications of the 12q13-14 region were identified in 6/8 tumours. Other, less consistent alterations were losses on 3p, 3q, 4q, 9p, 11q, 13q, Xp, and Xq, gains on 7p, 17p, and 17q as well as amplifications on 4q, 5p, 6p, and 11q [10]. Synovial sarcoma usually shows translocation of SYT-SSX t(X;18) (p11.2;q11.2) which is the characteristic and diagnostic feature.

Although aggressive surgery can offer dramatic palliation of symptoms caused by valvular and/or vascular obstruction, local recurrence and metastasis occur frequently and early, usually within 1 year [18]. Chemotherapy and radiation therapy have limited benefit [19]. Although surgery with negative resection margins is the only mainstay of successful treatment, complete tumour resection is possible in less than 50% of patients [4]. As life expectancy is twice as long for patients who undergo complete tumour resection [20], this indicates that early diagnosis and treatment have important prognostic and therapeutic implications.
